# Accelerating material property prediction using generically complete isometry invariants

**DOI:** 10.1038/s41598-024-59938-z

**Published:** 2024-05-02

**Authors:** Jonathan Balasingham, Viktor Zamaraev, Vitaliy Kurlin

**Affiliations:** Department of Computer Science, University of Liverpool, Liverpool, L69 3BX UK

**Keywords:** Computational methods, Computer science

## Abstract

Periodic material or crystal property prediction using machine learning has grown popular in recent years as it provides a computationally efficient replacement for classical simulation methods. A crucial first step for any of these algorithms is the representation used for a periodic crystal. While similar objects like molecules and proteins have a finite number of atoms and their representation can be built based upon a finite point cloud interpretation, periodic crystals are unbounded in size, making their representation more challenging. In the present work, we adapt the Pointwise Distance Distribution (PDD), a continuous and generically complete isometry invariant for periodic point sets, as a representation for our learning algorithm. The PDD distinguished all (more than 660 thousand) periodic crystals in the Cambridge Structural Database as purely periodic sets of points without atomic types. We develop a transformer model with a modified self-attention mechanism that combines PDD with compositional information via a spatial encoding method. This model is tested on the crystals of the Materials Project and Jarvis-DFT databases and shown to produce accuracy on par with state-of-the-art methods while being several times faster in both training and prediction time.

## Introduction

A solid crystalline material is made up of a periodically repeated unit cell containing a motif of atoms (ions or molecules). Crystals can distinguish themselves by atomic types (chemical elements and possibly charges of ions) and by the geometry of atomic centers. Both of these aspects can determine the various properties of a crystal. Knowledge of these properties is pertinent for determining whether a crystal can be experimentally synthesized or is useful for a particular application.

Determination of property values can be done using ab initio calculations with techniques like density functional theory (DFT)^1^. These techniques are often computationally expensive^2^. Further, they require extensive domain knowledge to be applied correctly, making them inaccessible. Recently, machine learning has become very popular as a substitute and has experienced success in decreasing computational costs while producing accurate predictions.

**Figure 1:**
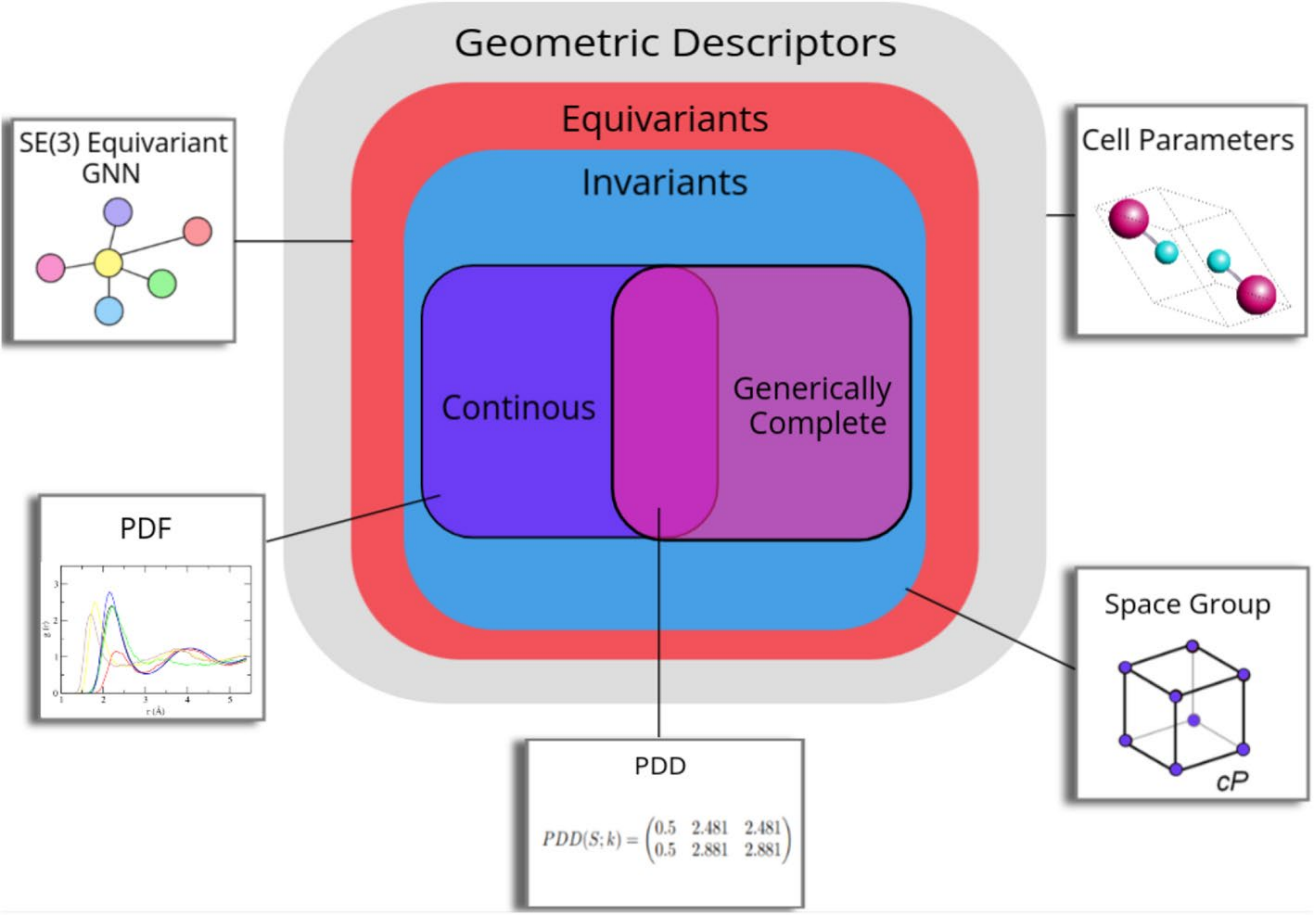
Classification of geometric descriptors for periodic crystals based on the properties possessed. Cell parameters consist of the unit cell lengths and angles, but this is ambiguous as there are an infinite number of unit cells. Space group is a label defined by the symmetry relations the crystal exhibits, but is sensitive to atomic perturbations. Equivariant GNNs cannot be used to distinguish periodic structures. PDF needs additional smoothing to retain continuity, introducing more parameters. The PDD is invariant, generically complete, and continuous under the EMD.

Any learning algorithm requires an input representation that adequately describes the object of interest. Objects similar to crystals, like molecules, are often treated as finite point clouds. This makes their representation more easily constructible than a representation for crystals, which are not bounded in size.

While a crystal can be described in several ways, descriptors that are easily human-interpretable, such as unit cell parameters or atomic coordinates are not useful for machine learning algorithms. Atomic coordinates do not retain invariance under rigid motion. Unit cell based descriptors are also ambiguous as there are infinitely many valid unit cells for a single structure. Such ambiguities can result in different model outputs for the same structure. Techniques such as data augmentation^3^ and parameter sharing^4^ can mitigate these effects but still do not guarantee the aforementioned consistency.

The *structure-property relationship*^5^ dictates that changes in the structure of a material result in changes in its properties. Distinction between crystals then allows for distinction between their respective property values. A machine learning algorithm (for a regression task) is a map from a crystal representation to value. If a representation cannot *distinguish* periodic crystals then two different crystals can incorrectly be perceived to be the same and so will the output property values. Similarly, if the same crystal can be represented in different ways, consistent mapping cannot be guaranteed.

The fundamental model of a crystal is a periodic set of points at all atomic centers (even without atomic types), see details in Definition 2.1. Since crystal structures are determined in a rigid form, their strongest practical equivalence is *rigid motion*, which is a composition of translations and rotations in $\mathbb {R}^n$. We consider a slightly weaker *isometry*, which is a composition of rigid motion and mirror reflections. Two periodic point sets *S* and *Q* are *isometric* if they are related by an isometry $f:\mathbb {R}^n\rightarrow \mathbb {R}^n$, so $f(S)=Q$. This work exclusively applies these concepts to dimension $n=3$.

An isometry *invariant* I is a property (descriptor or function) that is preserved under any isometry. The values of *I* should be simpler than the initial periodic set, for example, a scalar, vector, or matrix. Not all invariants can be considered equally useful, however. Space groups, for example, reflect the symmetry of a given material but tiny perturbations of atomic coordinates can change the material’s space group. Hence the important question is to quantify *how* different crystals are. Figure 1 illustrates the relationship between geometric descriptors based on their properties. A practically useful invariant should satisfy the following conditions introduced by^6^

### Problem 1

Find a function *I* on all periodic point sets in $\mathbb {R}^n$ subject to the following conditions:

- *Invariance*: If two periodic point sets, *S* and *Q* are isometric, then $I(S) = I(Q)$.
- *Generic Completeness*: If $I(S) = I(Q)$, then the two periodic sets are isometric.
- *Computability*: the invariant *I*, the metric *d*, and the reconstruction of *S* can be computed in polynomial time with respect to the size of the motif of any periodic point set *S*.
- *Reconstructability*: any periodic set *S* can be fully reconstructed from its invariant *I*(*S*).
- *Metric*: There exists a distance function *d* on the codomain *I* that satisfies the following: 1) $d(I(S), I(Q)) = 0$ iff $I(S) = I(Q)$. 2) $d(I(S), I(Q)) = d(I(Q), I(S))$. 3) For any three periodic sets *S*, *Q*,  and *T*, $d(I(S), I(Q)) + d(I(Q), I(T)) \ge d(I(S), I(T))$
- *Lipschitz continuity*: If a periodic set *Q* is obtained by shifting points within periodic set *S* by at most $\epsilon$, then the distance between the two periodic sets can be bound according to some distance function *d* such that $d(I(S), I(Q)) \le C\epsilon$ for some fixed constant *C*.

In the present work, an isometry invariant called the Pointwise Distance Distribution (PDD), defined formally in Definition 2.2, which has properties 1*a* and 1*c* (see Theorem 5.1 of^6^), and satisfies 1*b* and 1*d* for any periodic set in the general position^6^ along with sufficiently large *k*, and inclusion of the lattice (see theorem 4.4 of^6^). Conditions 1*e* and 1*f* (see Theorem 4.3 of^6^) are satisfied under the Earth Mover’s Distance (EMD) between PDDs.

The contribution of this work is a Transformer model^7^ which utilizes the PDD to make predictions on the properties of materials in a highly efficient manner compared to state-of-the-art models. In doing this, the gap between unambiguous crystal descriptors and machine learning models is bridged. Use of such a representation produces results on par or better than graph-based models, despite the additional structuring of data that comes with edges and edge embeddings. This model is faster in both prediction and training speed compared to two state-of-the-art models. To prove the method’s robustness, the model is applied to the crystals of the Materials Project^8^ and Jarvis-DFT^9^. Further experimentation on material classification, hyper-parameter sensitivity testing, and prediction of crystal properties without compositional information is included in the Supplemental Materials.

### Related work

Early works in crystal property prediction used more classical statistical methods like kernel regression^10^ before eventually moving towards deep learning^11^. More recent works have shifted to Graph Neural Networks (GNN)^12–21^ due to their ability to make use of structured data. Several of these focus on predicting the properties of the crystals contained within the Materials Project^8^ using a multigraph representation where vertices represent atoms and edges are embedded with the pairwise distances to an atom’s nearest neighbors. Some state-of-the-art models use line graphs to incorporate more geometric information like angles and dihedrals^13,22^. The derived line graphs can contain significantly more vertices and edges, incurring a higher computational cost. Other method^23^ take a physics principled approach and substitute the interatomic distances for interatomic potentials and capture a crystal’s periodicity using the infinite sum of these potentials.

While effective in modeling crystal structures, graphs are discontinuous under perturbations^24^. Small movements in the atomic positioning can cause significant changes to the graph’s topology. Some graphs are not unit cell invariant. Due to an infinite number of possible unit cells, the graph is then reliant on the data or the cell reduction technique used.

SE(3)-equivariant models such as Tensor Flow Networks^25^, SE(3)-Transformers^26^, and SE(3)-GNNs^27^ impose constraints on the set of learnable functions of the network such that the output is equivariant with respect to the input points. While effective on finite point clouds, they offer no promise of completeness with periodic point sets which is necessary for distinguishing between structures. These architectures can also be beneficial when predicting properties that are equivariant with respect to rigid motion, but the crystal properties examined here are invariant to such symmetries, and thus invariance of the model output through either the input or model architecture is required.

In addition to the properties mentioned earlier, the invariant needs to be able to be adapted for a learning algorithm. Further, it needs a way to incorporate compositional information as invariants typically only consider structure. Some invariants have been adapted for use in machine learning algorithms such as symmetry functions^28,29^ and Voronoi cells^30^. Both of these, however, still lack continuity. The Partial Radial Distribution Function is invariant and continuous but is not complete for homometric crystals. Smooth Overlapped Atomic Positions^31^ has been incorporated into models for property prediction, but is an invariant for atomic environments, not entire structures. Coulomb matrices use electrostatic interactions between atoms instead of Euclidean distances and are invariant and complete for molecules but have not been proven to retain these properties for the periodic case^32^. Average Minimum Distance (AMD)^33^ is invariant and continuous and has been used to predict lattice energies via Gaussian Process Regression^34^, but is incomplete and does not currently have a way to incorporate compositional information. The PDD has been used to derive a graph representation^35^, but this graph does not retain continuity.

## Methods

A periodic crystal can be represented as a periodic point set^36^ with points located at the atomic centers of the structure. They do not differentiate between the types of atoms and instead treat every point as unlabeled. A periodic point set (periodic set) can defined like so:

### Definition 2.1

(Periodic Point Set) For a set of *n* basis vectors ${\varvec{v}}_1 \ldots {\varvec{v}}_n \in \mathbb {R}^n$, the lattice *L* is formed by the integer linear combinations of these basis vectors $\{ \sum _{i=1}^n c_i {\varvec{v}}_i \vert c_i \in \mathbb {Z} \}$. The unit cell is the parallelepiped $U = \{ \sum _{i=1}^n t_i {\varvec{v}}_i | t_i \in [0, 1) \}$. For a unit cell *U*, the motif *M* is a finite subset of *U*. Then, a periodic point set *S* of lattice *L* and motif *M* is defined by $\{ \varvec{\lambda } + {\varvec{p}}: \varvec{\lambda } \in L , {\varvec{p}}\in M \}$.

The PDD of a periodic set is the $m \times (k+1)$ matrix where *m* is the number of atoms in the motif *M* and *k* is a positive integer indicating the number of nearest neighbors to use. Each row corresponds to a point in the motif and the entries within the row consist of the Euclidean distance to each of this point’s *k*-nearest neighbors within the entire periodic set *S*. The first entry of the row is assigned to be a weight equal to $\frac{1}{m}$ (the distances follow). Once the matrix is formed, rows that are the same are collapsed into a single row and their respective weights are added. Due to very small differences between rows caused by floating point arithmetic or atomic perturbations, it is common to use a tolerance, henceforth called the *collapse tolerance*, that allows rows with small non-zero differences (e.g. with respect to $L_\infty$ distance) to be treated as the same. By collapsing rows in the PDD, the resulting matrix representation is always the same for a given crystal, regardless of the unit cell. Formally,

### Definition 2.2

(Pointwise Distance Distribution) For a periodic set $S = L + M$ with a set of motif points $M = \{{\varvec{p}}_1, \ldots , {\varvec{p}}_m \}$ within a unit cell *U* of lattice *L*, the uncollapsed PDD matrix for a parameter $k \in \mathbb {N}^+$ is a $m \times (k+1)$ matrix where the $i^{th}$ row consists of the row weight $w_i = \frac{1}{m}$ followed by the euclidean distances $d_1 \ldots d_k$ from the point ${\varvec{p}}_i$ to its *k*-nearest neighbors such that $d_1 \le d_2 \ldots \le d_k$. If a group of rows is found to be identical (or close enough using a valid distance measure within some tolerance) then the matrix rows are collapsed and the weights of the involved rows are summed. The resulting matrix will then have less than *m* rows.

This matrix is referred to as $\textsc {PDD}(S;k)$ for a periodic set *S* and positive integer *k*.

### Periodic set transformer

In our model, rather than being considered a matrix of values, the PDD will be considered a set of grouped atoms. A single group of atoms corresponds to the *k*-nearest neighbor distances in a given row within the PDD matrix. Each member of the set will carry the weight provided by the row in the PDD. Any set *A* can trivially be turned into a weighted set by weighing each element by $\frac{1}{|A|}$. When the PDD is not collapsed, then there can be more than a single occurrence of any given element, making the uncollapsed PDD a multiset. Now, let *A* be a multiset of the form $A = \{ a_{i}^{(j)}: i \in [1, \dots , n], j\in [1,\dots , n_i] \}$ where $n_i$ is the multiplicity of element $a_i$ and $a_{i}^{(j)}$ is the $j^{th}$ occurrence of element $a_i$. This multiset can be turned into a weighted set by assigning each element $a_i$ with the weight $\frac{n_i}{n}$. We can recover the influence of multiplicity by the use of weights in our model.

**Figure 2:**
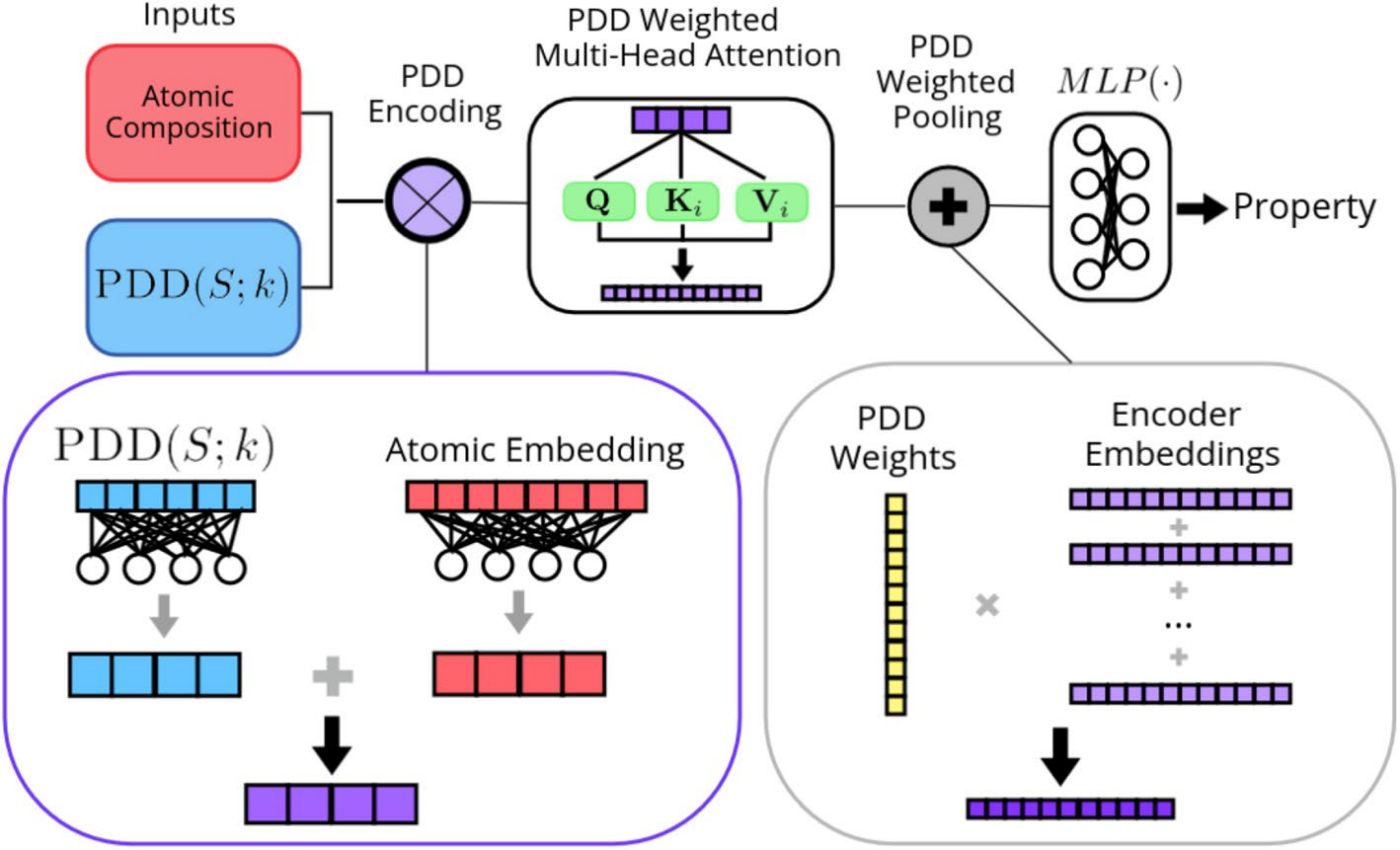
Overview of the architecture of the Periodic Set Transformer. PDD encoding is used to combine the structural information in the PDD with atomic types. The weights of the PDD are incorporated in the attention mechanism and during the pooling of the embeddings to define the multiplicity of the input set.

When a periodic crystal has its unit cell modified, the proportion of each atom is expanded or reduced. The use of weights captures this behavior in the form of a concentration or frequency.

We use an attention mechanism to find the interactions between members of the set. The rows of the PDD contain the pairwise distance information, but they do not indicate which atoms these distances correspond to. Application of the attention mechanism can help the model learn these interactions.

Let ${\varvec{R}}\in \mathbb {R}^{m \times k}$ be the PDD matrix containing *m* rows without the associated weight column. Let ${\varvec{w}}\in \mathbb {R}^{m \times 1}$ be the column vector containing the weights from the PDD matrix. The initial embedding is ${\varvec{X}}^{(0)} = {\varvec{R}}{\varvec{W}}_d$ where ${\varvec{W}}_d \in \mathbb {R}^{k \times d}$ is the initial trainable weight matrix. The embedding is updated according to:

1 $$\begin{aligned} {\varvec{X}}^{(1)} = {\varvec{X}}^{(0)} + SLP \Bigg ( \sigma \left( \frac{{\varvec{Q}}{\varvec{K}}^T}{\sqrt{d}} \right) {\varvec{V}}\Bigg ) \end{aligned}$$

where ${\varvec{Q}}= {\varvec{X}}^{(0)} {\varvec{W}}_Q$, ${\varvec{K}}= {\varvec{X}}_i^{(0)} {\varvec{W}}_K$ and ${\varvec{V}}= {\varvec{X}}^{(0)} {\varvec{W}}_{V}$ and *d* is the embedding dimension of the weight matrices for the query, key, and value ${\varvec{W}}_Q, {\varvec{W}}_K,$ and ${\varvec{W}}_V$ respectively as described in^7^. The function $\sigma$ is the softmax function with the PDD weights integrated into it; $\sigma$ is applied to each row ${\varvec{z}}$ of the input matrix, and *i* and *j* are used to index entries in ${\varvec{z}}$ and ${\varvec{w}}$. The $i^{th}$ entry of the output vector is defined by:

2 $$\begin{aligned} \sigma ({\varvec{z}})_i = \frac{w_i e^{z_i}}{\sum _{j=1}^{m} w_j e^{z_j}} \end{aligned}$$

The result is passed through a single-layer perceptron *SLP*. The layer normalization order described by^37^ is used for increased stability during training. Equation (2) describes the case for a single attention head. When multiple attention heads are used, the PDD weights are applied to each individually and the result is concatenated before being passed to the SLP like so:

3 $$\begin{aligned} {\varvec{X}}^{(1)} = {\varvec{X}}^{(0)} + SLP \Bigg ( \bigoplus _{i=1}^h \sigma \left( \frac{{\varvec{Q}}_i {\varvec{K}}_i^T}{\sqrt{d}} \right) {\varvec{V}}_i \Bigg ) \end{aligned}$$

where *h* is the number of attention heads, $\oplus$ is the concatenation operator and ${\varvec{Q}}_i, {\varvec{K}}_i$ and ${\varvec{V}}_i$ are the query, key and value for the $i^{th}$ head. This process is repeated *l* times; this determines the depth of the model. The embeddings are finally pooled into a single vector by reincorporating the PDD weights into a weighted sum of the row vectors ${\varvec{x}}_i$ of the final embedding ${\varvec{X}}^{(l)}$.

4 $$\begin{aligned} {\varvec{x}}= \sum _i w_i {\varvec{x}}_i \end{aligned}$$

This final embedding can be passed to a perceptron layer to predict the property value.

This version of self-attention can be applied to a weighted set or distribution. The weights are applied in such a way that the output of the PST is invariant to an arbitrary splitting of rows within the PDD. We provide a formal proof of this in Supplementary Material. An overview of the Periodic Set Transformer (PST) architecture with PDD encoding (described in the next section) can be seen in Fig. 2.

### PDD encoding

While structure is a powerful indicator of a crystal’s properties, there may be datasets in which it is not the primary differentiator of a set of crystals. In such cases, the composition of the atoms contained within the material has a heavy influence. The previously described transformer does a good job of utilizing the structural information within the PDD but does not provide an obvious way to include atomic composition.

Transformers for natural language processing tasks use positional encoding to allow the model to distinguish the position of words within a given sentence^38^. A recent transformer model, *Uni-Mol*^39^, which performed property prediction for molecules (among other tasks), used *3D spatial encoding* first proposed by^40^ to give the model an understanding of each atom’s position in space, relative to one another. This encoding is done at the pair level, using the Euclidean distance between atoms and a pair-type aware Gaussian kernel^41^. A transformer model for finite 3*D* points clouds is provided by^42^ via *vector* attention. The case for crystals is more difficult because they are not bounded in size and can exhibit many symmetries. Fortunately, by using the rows of the PDD we can distinguish each atom with structural information. We refer to this as *PDD encoding*.

When rows are grouped together, they are done so by having the same *k*-nearest neighbor distances. Though rare, it is possible for rows corresponding to different atom types to be collapsed. If this occurs, the selection of either atom type will result in information loss. To prevent this, we add the condition that the groups must be formed on the basis of having the same *k*-nearest neighbor distances and the same atomic species. In this case, the periodic point set has points that are labeled according to atomic type.

For a periodic set *S*, let *PDD*(*S*; *k*) be the resulting PDD matrix with parameter *k*. Let ${\varvec{R}}$ be *PDD*(*S*; *k*) without the initial weight column and ${\varvec{T}}$ be the matrix whose rows correspond to the vector of atomic properties used to describe the type of atom associated with each row of *PDD*(*S*; *k*). The initial set of embeddings for the attention mechanism is defined as ${\varvec{X}}^{(0)} = {\varvec{R}}{\varvec{W}}_s + {\varvec{T}}{\varvec{W}}_c$ where ${\varvec{W}}_s$ and ${\varvec{W}}_c$ are initial embedding weights. By starting with a linear embedding, the PDD row can be transformed to match the dimension of composition embedding. The parameter *k* used can then be changed as needed to include distance information from further neighbors.

## Results and discussion

### Prediction of materials project properties

The model will be applied to the data within the Materials Project. To make fair comparisons to other models we report the performance according to *Matbench*^43^, which contains data for various crystal properties. The error rates are reported using five-fold cross-validation with standardized training and testing sets for each fold. Further, tuning is done according to the models’ authors and thus our model can be compared to others more fairly.

The crystals in the Materials Project are highly diverse in composition. For all predictions, we include the composition of the crystal with PDD encoding. To incorporate this compositional information the *mat2vec* atomic embeddings supplied by^44^ are used. The embeddings have empirically been found to produce better performance than the one-hot encoded method used by CGCNN^45^. They also have the added convenience of not missing any atomic property information for certain elements.

In Table 1 we report the average mean-absolute-error (MAE) across the five test sets. We include the reported accuracies of other models to allow for comparison. The selection of models aims to present a high diversity in approaches while also coming from relatively recent publications. *CrabNet*^45^ is the only other Transformer model listed on Matbench. This model, in terms of architecture, is the most similar to the PST. Additionally, the atomic embeddings used to describe each chemical element are the same as those used in our model. The majority of models used in crystal property prediction use GNNs. Matbench features several of these, but the model that provides the best results on several properties is *coGN*. *coGN* is a GNN that includes angular and dihedral information through the use of line graphs. As such, the amount of information used is significantly more than the PST, which uses the distribution of pairwise distances. *Crystal Twins* (CT)^46^ is a model based on the convolutional layer developed by CGCNN^12^ (also used by several other models^16,18,47^) that uses self-supervised learning to create embeddings based on maximizing the similarity between augmented instances of a crystal.

**Table 1:** Five-fold cross-validation prediction MAE and standard deviation of MAE for properties of the crystals in the Materials Project.

Property	Units	PST	CrabNet	coGN	CrystalTwins
Formation energy	eV/atom	0.032 ± 0.0003	0.086 ± 0.001	**0.021 ± 0.0003**	0.037 ± 0.001
Band gap energy	eV	0.210 ± 0.002	0.266 ± 0.003	**0.156** ± **0.002**	0.264 ± 0.011
Shear modulus	$\textrm{log}_{10}\mathrm{(GPa)}$	0.074 ± 0.001	0.101 ± 0.002	**0.069 ± 0.001**	0.086 ± 0.004
Bulk modulus	$\textrm{log}_{10}\mathrm{(GPa)}$	0.056 ± 0.003	0.076 ± 0.003	**0.053 ± 0.003**	0.067 ± 0.003
Refractive index	n/a	**0.290 ± 0.078**	0.323 ± 0.071	0.309 ± 0.086	0.417 ± 0.080
Phonon peak	$1/\textrm{cm}$	**29.40 ± 1.40**	57.76 ± 5.73	29.71 ± 1.99	48.86 ± 7.69
Exfoliation energy	meV/atom	**31.15 ± 9.566**	45.61 ± 12.24	37.16 ± 13.68	46.79 ± 19.92
Perovskites FE	eV/cell	0.030 ± 0.001	0.406 ± 0.007	**0.027 ± 0.001**	0.042 ± 0.001

Bold values indicate the best (lowest) error rate while underlined values indicate the second-best error rate. PST performance is reported using PDD Encoding with a tolerance of $10^{-4}$ and $k=15$.

The PST and CrabNet share similarities in their construction. Both use *mat2vec* atomic embeddings and utilize a Transformer architecture with self-attention. While CrabNet uses fractional encoding to embed the multiplicity of each element type, we opt for the PDD-weighted attention mechanism and pooling described by Eqs. (2) and (4). The PST also uses PDD encoding to add structural information. This could also be done for CrabNet, but the combination of both fractional and PDD encoding is not guaranteed to aid in performance and simple summation of the encodings can cause ambiguities in the final embeddings, reducing performance. Across all properties the PST outperforms CrabNet, further indicating the usefulness of PDD encoding.

The performance disparity between the PST and coGN on formation and band gap energy can be difficult to discern. GNNs allow embeddings at the vertex and edge level. These embeddings can not only carry different information but allow for simultaneous updates to each of these embeddings, adding richness to the learned representation. CoGN takes this further and updates the original edges with message-passing from the derived line graph, allowing the inclusion of angular information. The edges of the line graph are further updated by its line graph, incorporating dihedral angles. These updates allow the model to learn a better representation of a crystal in latent space, which can be necessary for these larger datasets. This points to a current limitation for the PST. By using PDD encoding, we effectively limit opportunities for such updates to a single embedding representing both the atom’s properties and its structural behavior.

In Table 2 the time taken to train and test the PST and coGN are listed. Our model was trained for only 250 epochs while coGN is trained for 800 epochs. While this accounts for a large portion of the disparity, the time taken to train each model per epoch is also faster for our model in all properties except the refractive index. A batch size of 32 is used for datasets containing less than 5, 000 samples. For exfoliation energy and phonon peak, PST takes $68.1\%$ and $92.6\%$ of the time of coGN per epoch. The larger batch size of coGN allows it to have greater GPU utilization and thus, better training efficiency. For properties with greater than 5, 000 samples, the batch size for both models is the same. For these properties, the training time per epoch is between $65-70\%$ of coGN.

The prediction time disparity is more pronounced. For band gap and formation energy, the PST makes predictions approximately five times faster than coGN. Using nested line graphs introduces significant computational cost, but coGN is able to shrink the size of the graph by using the atoms in the asymmetric unit. The unit cell based approach was initially proposed by CGCNN^12^ and used in several follow-up works^13,16,18,46^, but the unit cell is inherently ambiguous and unnecessarily large in terms of the number of atoms needed to fully describe a crystal’s symmetry. The PDD at a collapse tolerance of exactly zero will have a number of rows less than or equal to the number of atoms in the asymmetric unit. At higher tolerances, this number will be reduced until reaching the number of unique chemical elements in the crystal.

**Table 2:** Single-fold prediction (measured in seconds) and training time (measured in minutes) for the PST (using $k=15$ and a collapse tolerance of $10^{-4}$) and coGN^22^ on Matbench^43^

Property	Samples	Training time (min.)		Prediction time (s.)	
		PST	coGN	PST	coGN
Formation Energy	132,752	159.1	772.1	2.79	15.25
Band Gap	106,113	126.6	602.1	2.38	11.88
Perovskites FE	18,928	13.41	62.37	0.31	2.93
Bulk Modulus	10,987	8.47	42.24	0.23	1.99
Shear Modulus	10,987	8.38	41.23	0.22	2.05
Refractive Index	4764	6.89	20.23	0.12	1.29
Phonon Peak	1265	1.81	6.25	0.04	0.87
Exfoliation Energy	636	0.89	4.18	0.02	0.79

Training and prediction was done using an Nvidia RTX 3090. Time does not include evaluation of the models on the validation sets or data pre-processing times.

#### Ablation study

In Table 3 we list the results for each “component” within the Periodic Set Transformer. In the row indicating PDD as the component, we train and test the model using only the structural information within the PDD. In the “Composition” component we pass the atomic encoding for the elements in the crystal and their concentration in the form of the PDD weights to the model without the PDD encoding. The model is run using $k = 15$ and a collapse tolerance of exactly zero.

By separating out each component of the model, we can interpret the importance of each to a particular property. Properties that experience a more significant decrease in performance when the PDD encoding is not used, can be ascribed to be more dependent on structural information. In all cases, the combination of both the composition and PDD encoding results in significantly lower error rates. We can conclude that this encoding method is effective in combining the structural and compositional information of a crystal structure.

**Table 3:** Effect of PDD encoding on prediction MAE of the Materials Project crystals.

Property (units)	Component MAE $\downarrow$		
	Composition	PDD	PST
Band gap eV	0.273	0.596	**0.212**
Formation eV/atom	0.088	0.421	**0.032**
Shear modulus $\textrm{log}_{10}\mathrm{(GPa)}$	0.107	0.132	**0.075**
Bulk modulus $\textrm{log}_{10}\mathrm{(GPa)}$	0.080	0.115	**0.055**
Refractive index	0.352	0.451	**0.292**
Phonon peak $1/\textrm{cm}$	50.39	74.71	**27.75**
Exfoliation meV/atom	46.91	39.35	**31.55**
Perovskites FE eV/cell	0.621	0.393	**0.030**

Results are separated by input components where “Composition” uses only the *mat2vec* atomic embeddings^44^ and “PDD” uses only the PDD. Errors in bold indicate the best performance and underlined errors indicate the second-best performance (lower is better $\downarrow$).

In Table 4 the effect of including the PDD weight in the attention mechanism described in Eq. (2) and in the pooling layer described by Eq. (4) is listed. A collapse tolerance of zero is used to remove any regularization effect (further described in the Supplemental Material).

The exclusion of weights from both the attention mechanism and pooling decreases accuracy significantly. Doing this removes all indications of multiplicity making discernment of crystals more difficult. The inclusion of the weights in the pooling layer is more impactful than when applied in the attention mechanism. The use of the weights in the pooling layer alone allows the model to perform better when the number of samples in the dataset is low. Datasets with fewer samples likely have less diversity amongst their crystals, making the need for recognizing the multiplicity of atoms less necessary.

**Table 4:** Effect of including the PDD weights as defined by Eqs. (2) and (4) on prediction MAE of the Materials Project crystals.

Property (units)	PDD weight inclusion MAE $\downarrow$			
	No weights	Attention only	Pooling only	PST
Band gap eV	0.278	0.244	0.219	**0.212**
Formation eV/atom	0.045	0.037	0.035	**0.032**
Shear modulus $\textrm{log}_{10}\mathrm{(GPa)}$	0.080	0.077	0.076	**0.075**
Bulk modulus $\textrm{log}_{10}\mathrm{(GPa)}$	0.059	0.059	0.056	**0.055**
Refractive index	0.314	**0.284**	0.288	0.292
Phonon peak $1/\textrm{cm}$	31.02	28.84	27.96	**27.75**
Exfoliation meV/atom	35.59	32.52	31.83	**31.55**
Perovskites FE eV/cell	0.031	0.030	0.031	**0.030**

Results for “No weights” use mean pooling and a normal softmax function. Errors in bold indicate the best performance and underlined errors indicate the second-best performance (lower is better $\downarrow$).

### Prediction of Jarvis-DFT properties

The *Jarvis-DFT* dataset^9^ is a commonly used set of materials with VASP^48^ calculated properties. The list of properties computed for the materials within the dataset is more extensive than that of the Materials Project. Its inclusion provides further evidence of the robustness of the model on an even wider variety of crystal properties.

The prediction MAE produced by PST and *Matformer* for 12 different properties from the dataset are included in Table 5. For Matformer, we retrain the model to ensure the training and testing sets are the same. We use the default parameters for the model defined by the authors’ codebase. We make one alteration to the training procedure; the number of epochs trained is reduced to 250. The number of epochs is the same as for our model.

**Table 5:** Prediction MAE on the properties of the Jarvis-DFT dataset using the PST and Matformer.

Property	Units	Samples	Test MAD	PST	Matformer
Formation energy	eV/atom	55,723	0.87	0.047	**0.033**
Band gap (OPT)	eV	55,723	0.99	0.172	**0.150**
Total energy	eV/atom	55,723	1.78	0.051	**0.036**
Ehull	eV	55,371	1.14	**0.052**	0.072
Bulk modulus	GPa	19,680	52.80	**10.76**	11.70
Shear modulus	GPa	19,680	27.16	**9.523**	10.13
Band gap (MBJ)	eV	18,172	1.79	**0.289**	0.304
Spillage	–	11,377	0.52	**0.367**	0.373
SLME (%)	–	9068	10.93	**4.61**	4.712
Max. piezo. stress coeff $(e_{ij})$	$\textrm{Cm}^{-2}$	4799	0.26	**0.127**	0.243
Max. piezo. strain coeff $(d_{ij})$	$\textrm{CN}^{-1}$	3347	24.57	**13.09**	18.03
Exfoliation energy	meV/atom	813	62.63	**30.91**	55.04

Results for Matformer^14^ are included for comparison. PST uses PDD encoding with $k=15$ and a collapse tolerance of $10^{-4}$. Bolded values indicate the best performance. The Mean-Absolute-Deviation (MAD) of the test set is included.

The PST outperforms Matformer in nine of the twelve properties tested. In particular, properties for which data is sparse yield results that favor the PST significantly (i.e. exfoliation energy, $e_{ij}$ and $d_{ij}$). Jarvis-DFT has two band gap values that are computed for its crystals, one which uses the optimized Becke88 functional (OPT)^49^ and the other uses the Tran-Blaha modified Becke Johnson potential (MBJ)^50^. The latter is more accurate (when compared to experimentally observed values) but also more computationally expensive. For this reason, there are significantly fewer computed values in the database. Interestingly, the PST produces a smaller error for the more accurate band gap values compared to Matformer, but a larger error for the less accurate OPT calculated values. A possible reason for this is the smaller sample size for which the PST has shown to be more effective. The disparity in performance for formation and total energy can be attributed to Matformer’s architecture which uses a GNN that updated both node and edge embeddings. This additional level of expression is helpful particularly when the size of the data grows larger, though it does come with added computational cost.

Matformer has been shown to produce even better results than the previous state-of-the-art model *ALIGNN*^13^ while taking roughly a third of the time to do both training and prediction. In Table 6, the training and prediction time for each of the properties in the Jarvis-DFT dataset is reported for the PST and Matformer. For the training time, the validation and pre-processing times are not included. The prediction time listed is the number of seconds taken to make predictions on the test set.

**Table 6:** Prediction (measured in seconds) and training time (measured in minutes) for the PST and Matformer^14^ on Jarvis-DFT datasets.

Property	Samples	Training time (min.)		Prediction time (s.)	
		PST	Matformer	PST	Matformer
Formation energy	55,723	41.36	345.8	0.329	29.77
Band gap (OPT)	55,723	41.62	343.9	0.347	29.86
Total energy	55,723	41.65	349.1	0.349	29.79
Ehull	55,371	40.69	348.9	0.352	28.93
Bulk modulus	19,680	14.12	93.33	0.135	11.12
Shear modulus	19,680	14.45	93.70	0.123	10.69
Band gap (MBJ)	18,172	13.38	118.7	0.107	9.71
Spillage	11,377	5.74	70.8	0.066	6.01
SLME (%)	9068	4.62	58.75	0.055	4.82
Max. piezo. stress coeff $(e_{ij})$	4799	3.52	23.15	0.029	2.57
Max. piezo. strain coeff $(d_{ij})$	3347	2.44	15.38	0.026	1.79
Exfoliation energy	813	0.63	5.30	0.008	0.41

Training and prediction was done using an Nvidia RTX 3090. Time does not include evaluation of the models on the validation sets or data pre-processing times.

In the closest training time comparison, the PST is still more than six times faster than Matformer. The training times for all properties fall between six and twelve times faster for the PST compared to Matformer. The performance increase can be attributed to several factors. Primarily, Matformer relies on a line graph (similar to coGN^22^) in order to update edge embeddings. While this increases the information used and leads to richer learned embeddings, the size of line graphs is considerably larger than the graph they are derived from. This, in turn, incurs a higher computational cost.

The difference in prediction times is more significant. Exfoliation energy is predicted over fifty times faster using the PST than with Matformer. This is the closest the two models perform to each other. Notably, exfoliation energy also has the fewest samples. For the bulk of the other properties, the speedup ranges between eighty and ninety times faster for the PST.

## Conclusion

The PDD is a generically complete and continuous invariant under isometry and permutations of points, hence independent of a unit cell. By using weights and creating a distribution, the PDD is able to represent an infinitely spanning object by its finite forms of behavior. Further, by collapsing rows in the PDD, the resulting representation can also be much smaller in comparison to the number of atoms within the unit cell, even when the cell is reduced.

The model is applied to the crystals of the Materials Project and Jarvis-DFT on a variety of material properties. Despite using less information in the model than more commonly employed graph-based models, the PST is able to produce results on par or even exceeding that of models like coGN and Matformer while taking significantly less time to train and make predictions.

### Supplementary Information

Supplementary Information.

## Data Availability

The data from the Materials Project is automatically downloaded through the code in the Github Repository. The Jarvis-DFT data can be downloaded through the Jarvis-Tools python package using the dft_3d_2021 database. Examples are included in the documentation here: https://pages.nist.gov/jarvis/databases/. The dataset for the crystals used in the lattice energy experiments is available at https://eprints.soton.ac.uk/404749/.

## Code availability

The code for the experiments is located at: https://github.com/jonathanBalasingham/Periodic-set-transformer. The code contains what is necessary to re-run the experiments done in “Prediction of Jarvis-DFT properties” and “Prediction of materials project properties” sections. It also contains the source code necessary to recreate the Figures included in the Supplemental Material. Details of how these plots are created are included in the Supplementary Material. The individual predictions for the Materials Project data are contained in *JSON* format within the repository. Proofs for the properties of the PDD mentioned in^51^, Problem 1.1 are included in the original paper^6^. More details on the actual implementation of the model, data pre-processing, and training are contained in Supplementary Material.
