# Supplementary material for: Accelerating material property prediction using generically complete isometry invariants
Source: Sci Rep. 2024 May 2;14:10132. doi: 10.1038/s41598-024-59938-z (PMC11065885; doi:10.1038/s41598-024-59938-z)
Supplement: Supplementary file 1 — Supplementary Information. [file 41598_2024_59938_MOESM1_ESM.pdf]

Accelerating Material Property Prediction using Generically Complete Isometry Invariants: Supplemental Material

Jonathan Balasingham[1,*], **Viktor Zamaraev**[1,*], **and Vitaliy Kurlin**[1,*]

[1]Department of Computer Science, University of Liverpool, Liverpool L69 3BX, UK

ABSTRACT

Periodic material or crystal property prediction using machine learning has grown popular in recent years as it provides a computationally efficient replacement for classical simulation methods. A crucial first step for any of these algorithms is the representation used for a periodic crystal. While similar objects like molecules and proteins have a finite number of atoms and their representation can be built based upon a finite point cloud interpretation, periodic crystals are unbounded in size, making their representation more challenging. In the present work, we adapt the Pointwise Distance Distribution (PDD), a continuous and generically complete isometry invariant for periodic point sets, as a representation for our learning algorithm. The PDD distinguished all (more than 660 thousand) periodic crystals in the Cambridge Structural Database as purely periodic sets of points without atomic types. We develop a transformer model with a modified self-attention mechanism that combines PDD with compositional information via a spatial encoding method. This model is tested on the crystals of the Materials Project and Jarvis-DFT databases and shown to produce accuracy on par with state-of-the-art methods while being several times faster in both training and prediction time.

1 Equivalence of Distributions in the PST

If a PDD is arbitrarily expanded or collapsed, the PST should produce the same results as these PDDs are considered equivalent. The same can be said of any input distribution. This is proven here:

Lemma 1.1. *Let A and B be weighted multisets each containing elements from the set $S = \{\boldsymbol{x}_1, \dots \boldsymbol{x}_n\}$. Each element $\boldsymbol{x}_i \in \mathbb{R}^{1\times n}$ occurs with multiplicity $m_i^{(a)} \in \mathbb{N}^+$ and $m_i^{(b)} \in \mathbb{N}^+$ in A and B respectively. Each element also carries weight $w_i^{(a)} \in \mathbb{R}^+$ and $w_i^{(b)} \in \mathbb{R}^+$ for A and B respectively. The application of the Periodic Set Transformer will yield equivalent output if*

$$w_i^{(a)} m_i^{(a)} = w_i^{(b)} m_i^{(b)}, \qquad \forall i \in \{1, \dots, n\} \tag{S1}$$

Proof. To prove that the output of the PST is equivalent for A and B, it is sufficient to prove that the output of σ (defined in Equation (3)) and the pooling layer (defined in Equation (4)) are the same for A and B. Let $\boldsymbol{q}_i = \boldsymbol{x}_i \boldsymbol{W}_Q$, $\boldsymbol{k}_i = \boldsymbol{x}_i \boldsymbol{W}_K$, and $\boldsymbol{v}_i = \boldsymbol{x}_i \boldsymbol{W}_V$ be the query, key, and value vectors produced by the weight matrices $\boldsymbol{W}_Q, \boldsymbol{W}_K, \boldsymbol{W}_V \in \mathbb{R}^{n\times d}$. First, note the pre-softmax attention weight from $\boldsymbol{x}_i$ to $\boldsymbol{x}_j$ can expressed as:

$$a_{ij} = \frac{\boldsymbol{q}_i \boldsymbol{k}_j^T}{\sqrt{d}} \tag{S2}$$

and is independent of both weight and multiplicity. Further, notice that the summation of an expression over A or B can be rewritten using its multiplicities. For A this is done like so:

$$\sum_{t=1}^{|A|} (\cdot) = \sum_{s=1}^{n} m_s^{(a)} (\cdot)$$

Using this equivalence, the function σ, used to calculate the attention weight from $\boldsymbol{x}_i$ to $\boldsymbol{x}_j$ for A and $\mathbb{B}$, is written as:

$$\alpha_{ij}^{(a)} = \frac{w_i^{(a)} exp(a_{ij})}{\sum_{k=1}^{n} w_k^{(a)} m_k^{(a)} exp(a_{ik})} \tag{S3}$$

and

$$\alpha_{ij}^{(b)} = \frac{w_i^{(b)} exp(a_{ij})}{\sum_{k=1}^{n} w_k^{(b)} m_k^{(b)} exp(a_{ik})} \tag{S4}$$

Using the condition provided by Equation (S1), Equation (S4) can be rewritten as:

$$\alpha_{ij}^{(b)} = \frac{w_i^{(b)} exp(a_{ij})}{\sum_{k=1}^{n} w_k^{(a)} m_k^{(a)} exp(a_{ik})} \tag{S5}$$

$$\sum_{k=1}^{n} w_k^{(a)} m_k^{(a)} exp(a_{ik}) = \frac{w_i^{(b)} exp(a_{ij})}{\alpha_{ij}^{(b)}} \tag{S6}$$

Substituting back into Equation (S3) gives us:

$$\alpha_{ij}^{(a)} = \alpha_{ij}^{(b)} \frac{w_i^{(a)} exp(a_{ij})}{w_i^{(b)} exp(a_{ij})} \tag{S7}$$

$$\alpha_{ij}^{(a)} = \alpha_{ij}^{(b)} \frac{w_i^{(a)}}{w_i^{(b)}} \tag{S8}$$

The attention weights produced from A can now be expressed in terms of the attention weights produced from B. The jth entry in the attention vector for $\boldsymbol{x}_i$ in A is:

$$y_{ij}^{(a)} = \sum_{j=1}^{n} \alpha_{ij}^{(a)} m_j^{(a)} v_{ji} \tag{S9}$$

$$= \sum_{j=1}^{n} \alpha_{ij}^{(b)} \frac{w_i^{(a)}}{w_i^{(b)}} m_j^{(a)} v_{ji} \tag{S10}$$

$$= \sum_{j=1}^{n} \alpha_{ij}^{(b)} \frac{w_i^{(a)}}{w_i^{(b)}} \left(\frac{w_i^{(b)} m_i^{(b)}}{w_i^{(a)}} \right) v_{ji} \tag{S11}$$

$$= \sum_{j=1}^{n} \alpha_{ij}^{(b)} m_i^{(b)} v_{ji} \tag{S12}$$

$$= y_{ij}^{(b)} \tag{S13}$$

Thus, the resulting embeddings from the attention mechanism are equivalent. While the embeddings themselves are equivalent, the cardinality for each embedding still differs according to each multisets' multiplicity. The pooling described by Equation (4) fixes this. Let $\boldsymbol{z}_i$ be the final embedding for $\boldsymbol{x}_i$. The output vector $\boldsymbol{z}$ from the pooling for A is the sum:

$$\boldsymbol{z}^{(a)} = \sum_{i=1}^{n} w_i^{(a)} m_i^{(a)} \boldsymbol{z}_i \tag{S14}$$

Substituting Equation (S1) again we get,

$$\boldsymbol{z}^{(a)} = \sum_{i=1}^{n} w_i^{(b)} m_i^{(b)} \boldsymbol{z}_i \tag{S15}$$

$$= \boldsymbol{z}^{(b)} \tag{S16}$$

Thus, the output of the PST for both A and B are equivalent. □

2 Details of MatBench and Jarvis-DFT Datasets

2.1 Materials Project

The crystals in the Materials Project[1] are acquired through the use of *MatBench*[2]. MatBench provides standardized training and test sets to ensure that property prediction models can be compared on equal footing. Each training and testing set of the five-fold cross-validation consists of 80% and 20% of the total data respectively. Validation is taken from the training set. In our application we use just 1% of the training data for validation. Due to the relatively simple architecture of the PST, overfitting a target property is more difficult and the typical procedure of selecting the model that performed the most accurately on the validation set is unnecessary. Instead, the PST is trained until the validation error converges.

In Table S1 we list the details of the MatBench dataset for the properties we tested. The information concerning the source of the data and how each of the properties for the crystals therein were calculated as follows.

- Formation Energy - Crystals from this dataset are sourced directly from the Materials Project[1]. The property is measured in $eV/atom$ and is calculated using DFT-GGA[3].
- Band Gap Energy - Crystals in this dataset are taken from the Materials Project[1]. The property is measured in eV and is calculated using DFT-GGA[3].
- Bulk/Shear Modulus - Crystals are sourced from the Materials Project. The value of the property is measured in $log_{10}(GPa)$. The elastic modulus tensor is derived from the full stress tensor which is calculated using the projector augmented wave method with DFT-GGA functionals[3,4].
- Perovskite Formation Energy - Crystals are taken from the work produced by Castelli et al.[5]. Property values are measured in $eV/unit\ cell$ and are calculated with DFT-GGA functionals using the RPBE approximation[6].
- Phonon Peak - Crystals are taken from the Materials Project. Property values are measured in $1/cm$ and are calculated using DFPT-GGA with PBEsol[7,8].
- Refractive Index - The crystals are taken from the Materials Project. Property values are derived directly from the dielectric tensor[9]. Dielectric tensors are calculated using Density Functional Perturbation Theory using VASP with PBE+U exchange-correlation functional[3,10] and Projector Augmented Wave psuedopotentials[4].
- Exfoliation Energy - Crystals are part of the Jarvis-DFT[11] *2D* materials dataset, but are originally sourced from the Materials Project and ICSD[12]. Property values are measured in $meV/atom$ and are calculated using DFT with optB88 functional[13]. Note, that these are different from the crystals mentioned below as part of the Jarvis-DFT dataset for exfoliation energy, which are part of the *3D* materials dataset.

Table S1. Details of the MatBench dataset by property, including the number of samples in each dataset and the mean-absolute-deviation (MAD).

Property	Units	Samples	MAD
Formation Energy	$eV/atom$	132,752	1.006
Band Gap Energy	eV	106,113	1.327
Shear Modulus	$log_{10}(GPa)$	10,987	0.293
Bulk Modulus	$log_{10}(GPa)$	10,987	0.290
Refractive Index	n/a	4,764	0.809
Phonon Peak	$1/cm$	1,265	323.787
Exfoliation Energy	$meV/atom$	636	67.202
Perovskites FE	$eV/cell$	18,928	0.566

2.2 Jarvis-DFT

Much of the Jarvis-DFT database consists of crystals taken from the Materials Project[1] and the ICSD[12]. After the initial structures are obtained, they are geometrically optimized using DFT, and then their properties are calculated. Details for each property are as follows:

- Formation Energy - The crystals in this dataset are taken from the Materials Project. Their values are measured in $eV/atoms$ and are calculated using OptB88vdW functional[14].

- Band Gap Energy (OPT) - The crystals in this dataset are taken from the Materials Project. The energy values are measured in eV and are calculated using the OptB88vdW functional[14,15].
- Total Energy - The crystals in this dataset are taken from the Materials Project and ICSD. Their values are measured in $eV/atom$ and are calculated using OptB88vdW functional[14].
- Bulk/Shear Modulus - The crystals are sourced from the Materials Project. Property values are measured in GPa and are calculated using the projector augmented wave method using the OptB88vdW functional[16].
- Band Gap Energy (MBJ) - The crystals are taken from the Materials Project. The property values are measured in eV and are computed using the modified Becke-Johnson potential[15,17].
- Spillage - Crystals are taken from the Materials Project. The structures are relaxed using the OptB88vdW functional but Spin-Orbit spillage is calculated using the PBE functional[18].
- SLME - Crystals are taken from the Materials Project. Spectroscopic Limited Maximum Efficiency (SLME) is calculated from the dielectric function and band gap (which are computed using DFT)[11].
- Maximum Piezoelectric Stress Coefficient (e_{ij}) - Crystals taken from the Materials Project. Value is measured in Cm^{-2} and is computed by taking the maximum entry from the Piezoelectric stress tensor.
- Maximum Piezoelectric Strain Coefficient (d_{ij}) - Crystals taken from the Materials Project. Property value is measured in CN^{-1} and is computed by taking the maximum entry from the Piezoelectric strain tensor.
- Exfoliation Energy - Property values are measured in $meV/atom$ and are calculated using DFT with optB88 functional[11]. These structures are different from the crystals mentioned as part of the MatBench dataset for exfoliation energy, which are part of the $2D$ materials dataset.

3 Applications of the Pointwise Distance Distribution

In the main text, we provided a formal definition for the PDD. Here, we will begin by providing an example of its construction.

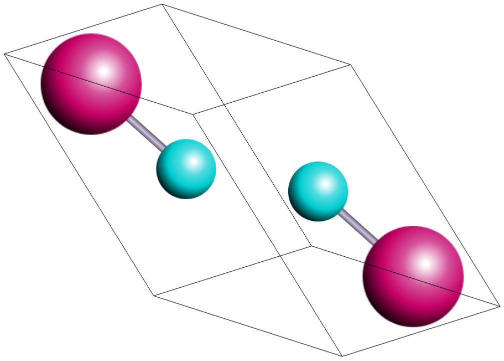

Figure S1. The unit cell of Lutetium-Silicon; Silicon is colored in teal and Lutetium in magenta.

Consider Lutetium-Silicon which is shown in Fig. S1. The unit cell pictured contains a total of four atoms, 2 of which are Lutetium and 2 of which are Silicon. If we use $k = 2$ nearest neighbors, the PDD matrix of this periodic set S before rows are grouped is

$$PDD(S;k) = \begin{pmatrix} 0.25 & 2.481 & 2.481 \\ 0.25 & 2.481 & 2.481 \\ 0.25 & 2.881 & 2.881 \\ 0.25 & 2.881 & 2.881 \end{pmatrix}$$

where the first column contains the weights for each row (atom). The second is the distance to the nearest neighbor and the third, is the distance to the second nearest neighbor. The first two rows are identical, as are the final two. Because of this, each of the two groups of rows can be grouped into a single row like so,

$$PDD(S;k) = \begin{pmatrix} 0.5 & 2.481 & 2.481 \\ 0.5 & 2.881 & 2.881 \end{pmatrix}$$

The rows are already lexicographically ordered so this is the finalized PDD. If a different unit cell is selected, this collapsing of matrix rows will continue to yield the same result as the proportion of each atom will not change.

The PDDs of two periodic crystals can be compared using the Earth Mover's Distance[19]. The weights of each PDD can be considered the distribution we are comparing in the minimum flow problem. We can visualize a set of crystals by projecting these distances onto a two or three-dimensional plane.

Subfigure S2a shows the projection of the pairwise distances of the PDDs of one hundred samples from each of the three crystals in this experiment using Multi-Dimensional Scaling (MDS). This technique attempts to map a set of pairwise distances to specific n-dimensional space by minimizing the difference between the actual pairwise distances and the distances between the points in the projected space. Even with this error, we are able to establish three distinct clusters in two-dimensional space according to their respective crystal type.

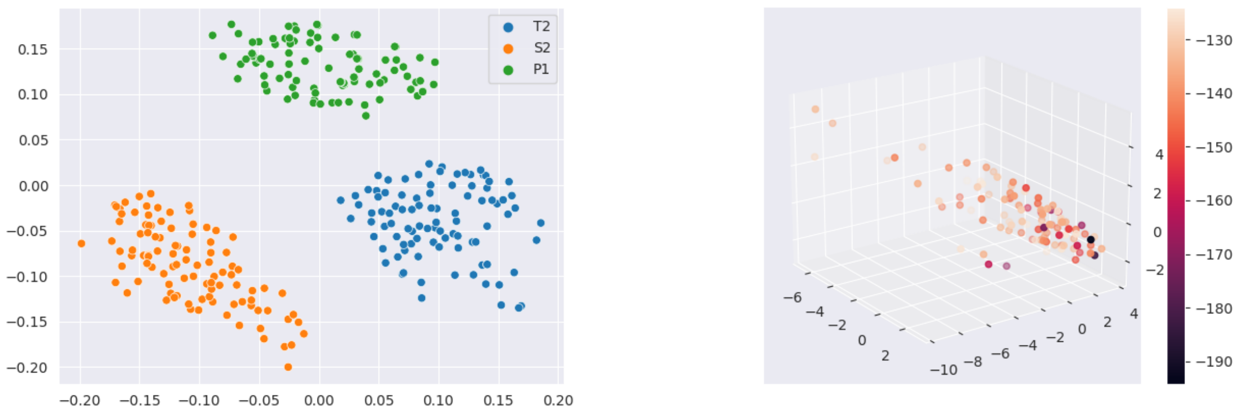

(a) MDS of sampled data by crystal type

(b) MDS of T2 crystals

Figure S2. Multi-dimensional scaling projection[20] on to $\mathbb{R}^2$ and $\mathbb{R}^3$ for the pairwise distances of crystals between each other. Subfigure (a) projects three types of crystals using distances created by using the Earth Mover's Distance between PDDs for $k = 10$. Subfigure (b) is created using the MDS projection of pairwise distances between PDDs at $k = 100$ for one hundred random samples from the T2 crystals colored by lattice energy measured in kJ/mol.

Subfigure S2b shows the projection of the pairwise distances between the crystals' PDD onto three-dimensional space for the T2 dataset. The colors of the points in each plot signify the lattice energy for the crystal. The distinction here is not as pronounced; this is to be expected as the sampled crystals are much more similar in their structure and thus the distances between the PDDs are smaller. Nonetheless, there is a discernible trend, and crystals that congregate near each other do share similar lattice energies.

4 Prediction of Lattice Energy

Here, the dataset considered contains simulated molecular crystals created by Pulido et al.[21] during the crystal structure prediction using quasi-random sampling[22]. This data is subsetted by the underlying molecule, e.g. T2. During structure prediction, crystals are generated while traversing the potential energy surface and their lattice energy is calculated using *DMACRYS*[23] to determine their stability.

Lattice energy is defined as the energy released during the crystallization of the constituent molecules into a lattice. Prediction of lattice energy is done in three different scenarios. In the first, the model will be applied to a single set of molecular crystals with the same composition using 80%/10%/10% training, validation, and testing splits. Next, the model is applied to multiple sets of crystals each with different underlying molecules using the same splits. The final experiment consists of the application of the model to a set of crystals with an underlying molecule it has not seen in training. The seen data is split 90%/10% for training and validation. To make predictions the PST is used with only the PDD as input and no knowledge of the composition.

Invariants are usually used to discern crystals by measuring differences between their structure. Here, the goal is to demonstrate the effectiveness of using an invariant as a representation for a machine learning algorithm. Even when compositional information is not present, the PDD can distinguish crystals with the EMD from the changes in the pairwise distances that

occur when the species of atoms are changed. Whether the same distinction can be made in the context of a learning algorithm has previously not been shown.

We make a comparison to another invariant that has been used to predict lattice energy. Average-minimum-distance[24] was used as input for a Gaussian regression model[25]. In Table S2 we list the performance on the test set of this AMD model to allow comparison between invariants.

Our model reduces the mean-absolute-error (MAE) rate by 21% compared to the Gaussian Process Regression technique which utilizes AMD[25]. The mean-absolute percentage error (MAPE) is also reduced by 1.63%. While we use $k = 60$ nearest neighbors for constructing the PDD, the AMD model uses $k = 500$ to achieve its best results. Despite the use of this additional information, the model using the PDD still performs more accurately.

Table S2. Results of lattice energy prediction using the PDD with the PST and AMD with Gaussian regression on three different tasks using 80%/10%/10% training, validation, and testing splits. **(a)** uses only experimentally generated structures of the T2 molecular crystal based on triptycene. **(b)** adds two additional sets of molecular crystals, P1 (based on pentiptycene) and S2 (based on spiro-biphenyl). **(c)** uses P1, P1M (methylated analogue of P1) and P2 (benzimidazolone series pentiptycene) in the training and validation set using a 90%/10% split. The test set consists only of P2M, the methylated analogue of P2.

	Train	Train Size	Test	Test Size	Invariant	MAE (kj/mol) ↓	MAPE ↓
**(a)**	T2	4,630	T2	578	AMD	4.79	4.31%
					PDD	3.76	2.68%
**(b)**	T2, P1, S2	14,547	T2, P1, S2	1,819	AMD	4.68	2.83%
					PDD	4.11	2.52%
**(c)**	P1,P1M,P2	22,995	P2M	7,352	AMD	12.99	6.89%
					PDD	7.24	3.89%

In the second row of Table S2 we list the results of the second experiment. The previous task is extended to a dataset of crystals that contains different underlying molecules. These molecules have different compositions. This compositional information is not contained within the PDD (and thus, not in our input). The model will have to discern crystals solely by their structure. While the overall MAE has increased slightly, the percentage error has decreased. The domain on which the lattice energies lie is different for each type of crystal. Using the PDD alone is enough for the algorithm to distinguish the crystal types and predict lattice energy accordingly.

The final experiment uses the data from the P1, P1M, and P2 crystals in the training and validation data. The test set consists of the P2M crystal, which is not part of the either training or validation set. This experiment is the closest to real-world conditions in which new crystals often arise and finding the stable forms is crucial, but information on their lattice energy is unavailable.

When lattice energy is calculated using ab initio calculations, the range of the energies varies from crystal to crystal. When introducing a new type of crystal for our algorithm to make predictions on, this can become a problem as extrapolation to unvisited parts of the lattice energy range can result in high error rates. Fortunately, lattice energies between different types of crystals, are not usually meant to be compared. Instead, they are generated for potential polymorphs of a crystal in an effort to find those with the highest stability for synthesis. We can make use of this fact when applying our model to novel crystals. It is not necessary to predict the correct range of lattice energies; instead, the model needs to be able to predict the lattice energies of the various structures such that their ordering according to their lattice energy is correct. This task could feasibly be turned into a *learning-to-rank* problem[26], but as a regression task, it allows for a more general approach since the predicted lattice energies can be ordered after the fact.

Each dataset has its lattice energies shifted by the mean lattice energy towards zero. By doing this they each maintain their distribution but now overlap around the origin. The model is trained and validated based on this shifted data. The MAE of the predictions on the test set after they have been shifted back is 7.24 kJ/mol and the MAPE is 3.89%.

While the MAE and MAPE are higher than in previous experiments, the improvement over AMD is more significant. The majority of errors come from underestimating the lattice energy. The datasets tend to grow sparser in these areas where lattice energy is lower as this is where the most stable structures tend to lie. Having a false positive (predicting a higher energy structure to be lower) increases the number of potentially stable structures. False negatives are more impactful as they may result in a structure not being considered entirely due to its seemingly low stability.

The histogram in Fig. S3a shows the lattice energies (in kJ/mol) of the three crystals within the dataset. Figure S3b shows the comparison of the predictions of the model against the true property values in the dataset, referred to as ground truth values.

Predictions that have lower errors will have their point placed closer to the line colored in blue. The bulk of the points share their error both below and above the true lattice energy as we would expect in a model without bias. There are a few outliers, in particular, a single crystal from the T2 dataset has a predicted lattice energy of just -0.41. Prevention of such errors can be

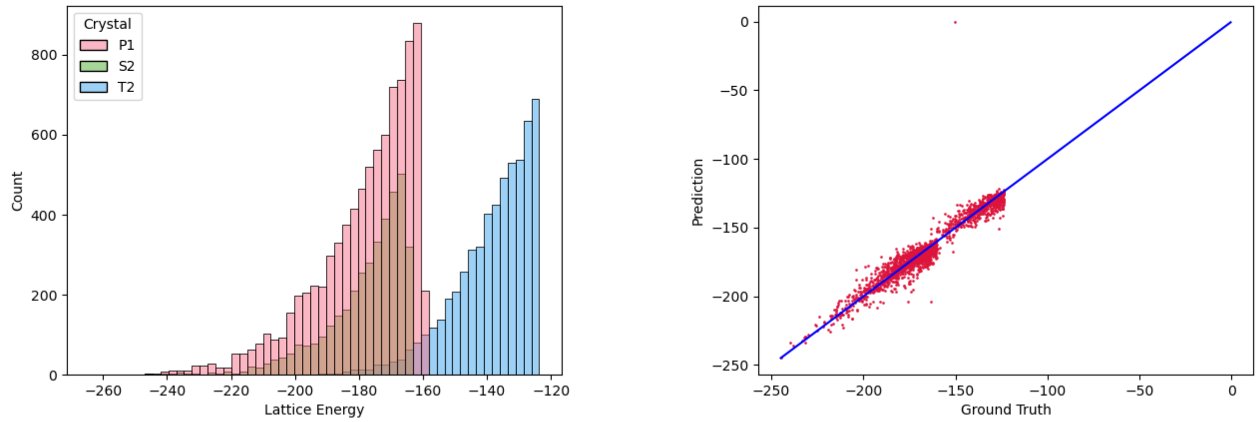

(a) Distribution of ground truth lattice energies.

(b) Ground truth lattice energy vs. predictions

Figure S3. (a) The distribution of the lattice energies of the T2, S2 and P1 crystals. (b) The predictions of T2, S2, and P1 compared to the ground truth lattice energies in kJ/mol.

mitigated by using a different loss function than MAE. In particular, mean-squared error (MSE) and Huber loss can hedge against outlying errors by increasing their contribution to the loss function. We choose to not present the results using these loss functions as MAE still provides better results for MAE and MAPE.

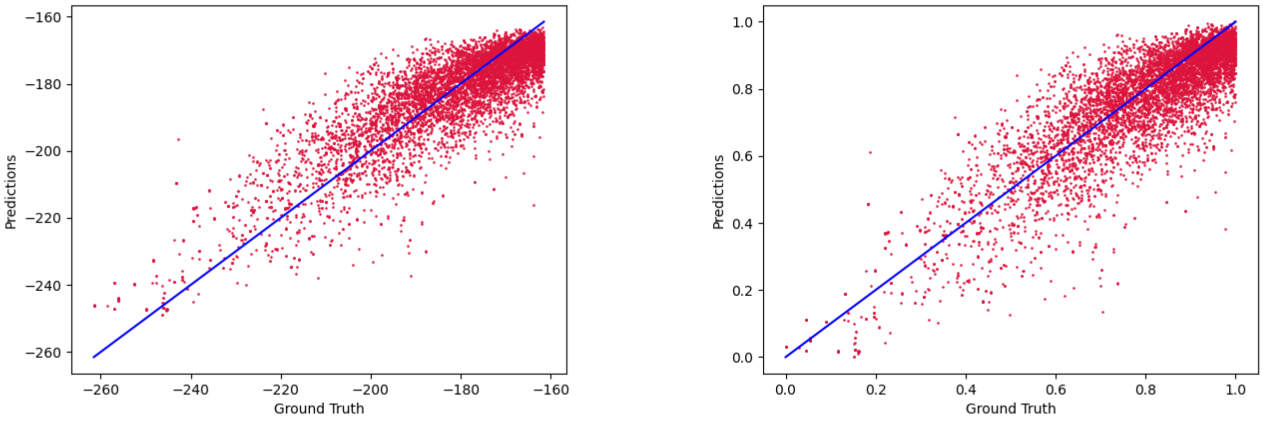

(a) Ground Truth vs. Predictions re-scaled according to mean.

(b) Ground Truth vs. Predictions normalized between zero and one.

Figure S4. (a) Comparison of predictions vs. true (ground truth) values of the test set after the predictions are scaled back by the mean of the lattice energies. (b) The comparison of predictions vs. ground truth after both have been normalized between zero and one.

Figure S4a shows the plot of the original predictions on P2M after they have been re-scaled back away from the mean lattice energy. Figure S4b shows the re-scaled predictions and the true property values found in the dataset (henceforth, referred to as ground truth values) to between zero and one. By doing this, we can see the ordering of the predictions compared to the true lattice energies. The scatter plot in Fig. S4b allows us to compare lattice energies relative to other predictions.

If the error rate produced by the final experiment of section 4 is inadequate, we can supplement the training with predictions from classical methods. For a novel crystal, ab initio calculations can be used to generate lattice energies for a small subset of structures. These samples can be integrated into the training set to improve the overall results. In order to make this practical, we can only produce lattice energies for a limited portion of structures; we limit this to 10% (or under) of the total structures.

Table S3 displays the result of adding this supplemental data to the training set. As expected, the addition of data decreases

Table S3. Effect of including portions of the P2M data into the training set on prediction accuracy.

Samples	Percent of P2M Data	Test MAE (kj/mol) ↓	Test MAPE ↓
0	0%	7.24	3.89%
367	5%	5.82	3.17%
735	10%	5.50	3.02%

MAE and MAPE. Even with as few as 367 samples (or 5% of the total dataset), the reduction in error is significant. This process experiences diminishing returns as the amount of the original dataset is used in the training set. Though the MAE and MAPE continue to decrease, it is questionable whether or not spending time generating the instances using classical methods is worth the additional performance gains.

5 Additional Experiments

5.1 Identifying Crystals with Outlying Property Values

The properties that have been targeted with the PST are all regression tasks. These can also be reframed as a classification task wherein the model attempts to identify if a given material has a "high" (or alternatively, a "low") property value. Due to the ambiguity of this term, a threshold at which a property qualifies as having a "high" value needs to be established. In the following experiments, a value exceeding the 90th percentile of the test set is considered "high".

MAE is no longer a suitable metric for measuring the model's performance. Instead, four other metrics are used: accuracy, recall, and precision. We define the term *true positive* (TP) as the correct classification of materials with a "high" property value. *False positive* (FP) is the incorrect classification of a sample with a "high" property value. *True negative* (TN) refers to the correct classification of a crystal that does not have a "high" property value. Finally, *False Negative* refers to the incorrect classification of material that does not have a "high" property value. Accuracy is determined by the expression $(TP+TN)/(TP+TN+FP+FN)$. Precision is found by evaluating the expression $TP/(TP+FP)$ and recall is found using $TP/(TP+FN)$. The results of this classification task for all properties provided by MatBench are listed in Table S4. The scores listed are the average across all five folds of each dataset.

Table S4. Results of the classification of materials with property values in the 90th percentile on the MatBench datasets. Threshold refers to the value at which a crystal is determined to be a positive sample if its own property value is higher. The abbreviations in the Table are as follows: (TP) True Positive, (FP) False Positive, (FN) False Negative, (TN) True Negative, and (Acc.) Accuracy.

Property	Threshold	TP	FP	FN	TN	Acc. (%)	Recall (%)	Precision (%)
Formation Energy	-0.015 $eV/atom$	12,309	966	633	118,844	98.8	95.1	92.7
Band Gap Energy	3.69 eV	9,489	1,126	1,124	94,374	97.9	89.4	89.4
Shear Modulus	1.99 $log_{10}(GPa)$	877	204	179	9,772	96.5	83.1	81.1
Bulk Modulus	2.29 $log_{10}(GPa)$	955	132	135	9,765	97.6	87.6	87.9
Refractive Index	3.392	386	94	78	4,206	96.4	83.2	80.4
Phonon Peak	1,128 $1/cm$	123	7	8	1,127	98.8	93.4	94.6
Exfoliation Energy	204.4 $meV/atom$	45	20	18	553	94.0	71.4	69.2
Perovskites FE	2.436 $eV/cell$	1,820	50	78	16,890	99.3	95.9	97.3

The accuracy for all properties is relatively high, with a lower bound of 94% occurring for the property with the fewest number of samples: exfoliation energy. The trend is similar for recall and precision, with properties containing a smaller sample size having lower scores. Across all properties, the relationship between precision and recall remains balanced with no single property containing an exceedingly high rate of false negatives or false positives.

Increasing the threshold at which crystals are classified as positive decreases both recall and precision, but accuracies increase. This can be seen in Table S5 where the threshold is increased to the 95th percentile. At this level, most properties still perform well with only small reductions in recall and precision. Exfoliation energy is an exception to this. This is the byproduct of having a comparatively small dataset.

If materials with property values above the 99th percentile are considered positive samples the precision and recall of refractive index drops to 24% and 14% percent respectively. Notably, at this threshold, there are only 7 such crystals which qualify as positive. Datasets with a large number of crystals do not experience as drastic a decrease in efficacy. Band Gap energy, for example, experiences a reduction to 87.1% and 80.4% for precision and recall, respectively.

Table S5. Results of the classification of materials with property values in the 95th percentile on the MatBench datasets. Threshold refers to the value at which a crystal is determined to be a positive sample if its own property value is higher. The abbreviations in the Table are as follows: (TP) True Positive, (FP) False Positive, (FN) False Negative, (TN) True Negative, and (Acc.) Accuracy.

Property	Threshold	TP	FP	FN	TN	Acc. (%)	Recall (%)	Precision (%)
Formation Energy	0.178 $eV/atom$	6,015	625	380	125,732	99.2	94.1	90.6
Band Gap Energy	4.55 eV	4,662	648	604	100,199	98.9	88.6	87.8
Shear Modulus	2.08 $log_{10}(GPa)$	408	128	98	10,353	98.0	81.0	76.1
Bulk Modulus	2.35 $log_{10}(GPa)$	465	73	83	10,366	98.6	84.9	86.4
Refractive Index	4.353	168	72	65	4,459	97.1	72.1	70.0
Phonon Peak	1,428 $1/cm$	62	3	8	1,192	99.1	88.6	95.4
Exfoliation Energy	318.0 $meV/atom$	17	18	9	592	95.8	65.4	48.6
Perovskites FE	2.891 $eV/cell$	887	40	38	17,963	99.6	95.9	95.7

5.2 Effect of k-nearest neighbors

PDD encoding can be said to be parameterized by two values, the collapse tolerance and the number of k-nearest neighbors. The integer k determines the dimensionality of initial PDD encoding embedding. As k increases, it retains all information from the previous (smaller) values of k. The initial nearest neighbor distances are the most important and the embedding has diminishing returns after this. For any value of $k > 1$, the PDD is invariant. Thus, if the PDD is different, the crystals are guaranteed to be structurally different. In order for the PDD to be distinct such that if any two crystals are different, their PDDs are different, we need the property of generic completeness. This property is given provided the lattice L and sufficiently large k. An upper bound on this k is when all distances in the last column of the PDD are larger than twice the covering radius of the lattice L of the periodic set. We would expect the performance of the model to increase up until this point.

The upper bound on k can be exceedingly large so we implement a heuristic to find a lower bound that is more computationally efficient. As k increases, the number of rows collapsed in the PDD will either stay the same or decrease. The lower bound on k can be considered an integer large enough that the groups established at the upper bound of k are the same as this lower bound. Each crystal could have a different k for which this requirement is met. Our encoding method prohibits the use of a dynamic k value, therefore we need a consistent value for k that can be applied to all crystals in the dataset. The results in Table 1 use $k = 15$. At this value of k the previously mentioned lower bound is satisfied for 99.1% within the formation energy dataset. Increases in k past this point cause marginal improvements to this coverage that were deemed insufficient when the increased computational cost is considered.

Property (units)	MAE by k-Nearest Neighbors PDD Encoding ↓			
	5	10	15	20
Band Gap eV	0.261	0.232	**0.212**	<u>0.214</u>
Formation $eV/atom$	0.039	0.034	<u>0.032</u>	**0.032**
Shear Modulus $log_{10}(GPa)$	0.087	0.077	<u>0.075</u>	**0.075**
Bulk Modulus $log_{10}(GPa)$	0.064	0.059	<u>0.055</u>	**0.056**
Refractive Index	0.318	0.291	<u>0.292</u>	**0.283**
Phonon Peak $1/cm$	**26.38**	27.89	<u>27.74</u>	29.21
Exfoliation $meV/atom$	38.77	33.63	**31.55**	<u>31.70</u>
Perovskites FE $eV/cell$	0.031	0.031	<u>0.030</u>	**0.030**

Table S6. Prediction MAE on the Materials Project crystals for various k-nearest neighbors PDD Encoding at a collapse tolerance of exactly zero. Errors in bold indicate the value of k with the best performance and underlined errors indicate the second-best performance (lower is better ↓).

The error rates listed in Table S6 vary the value of k and report the resulting mean MAE across the five folds. As expected, the lower values of k generally result in higher MAE. Increasing k eventually causes the error rates to stop decreasing. This is also in line with what would be expected as the PDD has enough information to distinguish itself and additional distances are unnecessary. This is not the case only with phonon peak, however, the differences in MAE are relatively small when the deviation of the errors across the folds is considered.

5.3 Effect of collapse tolerance

The collapse tolerance dictates which rows of the PDD will be collapsed. As this parameter increases, the size of the grouped rows will increase. Once rows are grouped, their distances are averaged in the row which represents the group. The change in this averaged row is proportional to the size of the collapse tolerance. In $PDD(S;k)$, as the collapse tolerance approaches infinity, the PDD will decrease in the number of rows until it consists of just a single row with a weight equal to one. In $PDD(S;k)$, the same increase in collapse tolerance will result in a number of rows within the PDD equal to the number of unique elements within the crystal. In both cases, a collapse tolerance that is large enough will result in information loss, eventually increasing errors in predictions.

Property (units)	MAE by Collapse Tolerance in PDD Encoding ↓			
	1.0	10^{-2}	10^{-4}	0.0
Band Gap eV	0.241	0.222	<u>0.210</u>	**0.212**
Formation $eV/atom$	0.037	0.033	<u>0.032</u>	**0.032**
Shear Modulus $log_{10}(GPa)$	**0.074**	0.074	<u>0.074</u>	0.075
Bulk Modulus $log_{10}(GPa)$	0.056	0.056	**0.056**	<u>0.055</u>
Refractive Index	**0.283**	0.292	<u>0.290</u>	0.292
Phonon Peak $1/cm$	<u>28.41</u>	28.50	29.40	**27.74**
Exfoliation $meV/atom$	32.19	**31.13**	<u>31.15</u>	31.55
Perovskites FE $eV/cell$	0.030	0.030	0.030	0.030

Table S7. Prediction MAE on the Materials Project crystals using PDD Encoding at various collapse tolerances with $k = 15$. Errors in bold indicate the collapse tolerance with the best performance and underlined errors indicate the second-best performance (lower is better ↓).

The collapse tolerance is varied and then applied to the Materials Project crystals. The results of this experiment are listed in Table S7.

By increasing the collapse tolerance and reducing the number of rows within the PDD, we can increase the speed of computations. Thus, it is important to choose a tolerance that is maximal, while not sacrificing accuracy. The impact of the collapse tolerance on the size of representation is listed in Table S8. These values are calculated by dividing the number of rows in the PDD by the number of atoms in the unit cell. The number of atoms in the unit cell is used to determine the number of vertices in the crystal graph[27]. In this way, the size of our representation can be compared to that of popular graph-based models. Data for crystals typically comes in the form of Crystallographic Information Files (CIF). These files also indicate the amount of potential measurement error for the atomic positions. This is used as a guide and a collapse tolerance of 10^{-4} is used in the experiments for the results in Table 1. Sometimes, however, a higher collapse tolerance can act as a regularization technique that is useful on smaller datasets. This effect is seen in the results for refractive index and exfoliation energy, but there is still a balance to be struck. In larger datasets, the performance regression is more noticeable. A collapse tolerance of one is far higher than what would be necessary to cover measurement error in atomic coordinates and would not be advised, even with the potential efficiency gains. Overall, the collapse tolerance does not have a very large impact due to the prevention of rows corresponding to different atoms from being collapsed in the PDD.

Property	Mean $\|M\|$	Size of Input				Percentage of $\|M\|$			
		0.0	10^{-4}	10^{-2}	1.0	0.0	10^{-4}	10^{-2}	1.0
Phonon Peak	7.5	7.3	3.6	3.5	3.4	96.8%	55.7%	54.5%	53.8%
Ref. Index	16.9	16.5	6.7	6.2	5.8	97.7%	49.1%	46.3%	44.1%
Bulk Modulus	8.6	8.3	4.1	3.9	3.7	96.7%	62.1%	60.4%	59.4%
Shear Modulus	8.6	8.3	4.1	3.9	3.7	96.7%	62.1%	60.4%	59.4%
Band Gap	30.0	29.2	13.1	12.0	10.1	97.0%	52.0%	48.5%	44.2%
Formation	29.1	28.4	12.7	11.6	9.9	96.9%	53.1%	49.7%	45.7%
Exfoliation	7.2	7.1	3.6	3.3	3.2	98.8%	55.9%	51.9%	51.5%
Perovskites	5.0	4.9	4.6	4.6	4.6	99.0%	94.8%	94.8%	94.8%

Table S8. Size of the input representation for each dataset in the Materials Project at various collapse tolerances at $k = 15$ compared to the number of atoms in the unit cell $|M|$. The size of the input refers to the cardinality of the input set determined by the number of rows in the PDD. The percentage of $|M|$ is the input set's cardinality divided by the number of atoms in the unit cell, expressed as a percentage.

6 Implementation Details

The Periodic Set Transformer is implemented using *PyTorch*[28]. There is also a version implemented using *Tensorflow*[29], however, we have found this version to significantly underperform when compared to the PyTorch version. We believe this to be due to how the output of individual attention head output is handled in their respective implementations of Multi-head Attention.

Data pre-processing is fairly minimal. Each crystal comes in the form of a `Pymatgen` structure. The structure is converted into a `PeriodicSet` object. This functionality is provided by the *AMD* package[24]. The PDD of each of the `PeriodicSet` objects is then calculated with the desired collapse tolerance and k value. Each column is then normalized to between zero and one. This is not necessary for achieving the desired accuracy but it does significantly improve the speed of training by requiring fewer epochs.

With respect to the results in Table 1, training is done on each property with the same hyper-parameters. The hyper-parameters that govern PDD encoding remain the same for all properties: a tolerance of 10^{-4} and $k = 15$. Training options including weight decay, epochs, and learning schedule are kept the same as well, except for batch size. Batch size is either 32 or 64 depending on the number of samples: 32 if the number of crystals in the dataset is less than 5000 and 64 if greater.

References

1. Jain, A. *et al.* The Materials Project: A materials genome approach to accelerating materials innovation. *Appl. Phys. Lett. Mater.* **1**, 011002, DOI: 10.1063/1.4812323 (2013).
2. Dunn, A., Wang, Q., Ganose, A., Dopp, D. & Jain, A. Benchmarking materials property prediction methods: the matbench test set and automatminer reference algorithm. *npj Comput. Mater.* **6**, 138 (2020).
3. Perdew, J. P., Burke, K. & Ernzerhof, M. Generalized gradient approximation made simple. *Phys. review letters* **77**, 3865 (1996).
4. Blöchl, P. E. Projector augmented-wave method. *Phys. review B* **50**, 17953 (1994).
5. Castelli, I. E. *et al.* New cubic perovskites for one-and two-photon water splitting using the computational materials repository. *Energy & Environ. Sci.* **5**, 9034–9043 (2012).
6. Hammer, B., Hansen, L. B. & Nørskov, J. K. Improved adsorption energetics within density-functional theory using revised perdew-burke-ernzerhof functionals. *Phys. review B* **59**, 7413 (1999).
7. Petretto, G. *et al.* High-throughput density-functional perturbation theory phonons for inorganic materials. *Sci. data* **5**, 1–12 (2018).
8. Perdew, J. P. *et al.* Restoring the density-gradient expansion for exchange in solids and surfaces. *Phys. review letters* **100**, 136406 (2008).
9. Petousis, I. *et al.* High-throughput screening of inorganic compounds for the discovery of novel dielectric and optical materials. *Sci. data* **4**, 1–12 (2017).
10. Dudarev, S. L., Botton, G. A., Savrasov, S. Y., Humphreys, C. & Sutton, A. P. Electron-energy-loss spectra and the structural stability of nickel oxide: An lsda+ u study. *Phys. Rev. B* **57**, 1505 (1998).
11. Choudhary, K. *et al.* The joint automated repository for various integrated simulations (jarvis) for data-driven materials design. *npj computational materials* **6**, 173 (2020).
12. Hellenbrandt, M. The inorganic crystal structure database (icsd)—present and future. *Crystallogr. Rev.* **10**, 17–22 (2004).
13. Choudhary, K., Kalish, I., Beams, R. & Tavazza, F. High-throughput identification and characterization of two-dimensional materials using density functional theory. *Sci. reports* **7**, 5179 (2017).
14. Klimeš, J., Bowler, D. R. & Michaelides, A. Chemical accuracy for the van der waals density functional. *J. Physics: Condens. Matter* **22**, 022201 (2009).
15. Choudhary, K. *et al.* Computational screening of high-performance optoelectronic materials using optb88vdw and tb-mbj formalisms. *Sci. data* **5**, 1–12 (2018).
16. Choudhary, K., Cheon, G., Reed, E. & Tavazza, F. Elastic properties of bulk and low-dimensional materials using van der waals density functional. *Phys. Rev. B* **98**, 014107 (2018).
17. Tran, F. & Blaha, P. Accurate band gaps of semiconductors and insulators with a semilocal exchange-correlation potential. *Phys. review letters* **102**, 226401 (2009).

18. Choudhary, K., Garrity, K. F. & Tavazza, F. High-throughput discovery of topologically non-trivial materials using spin-orbit spillage. *Sci. reports* **9**, 8534 (2019).

19. Rubner, Y., Tomasi, C. & Guibas, L. J. The earth mover's distance as a metric for image retrieval. *Int. journal computer vision* **40**, 99 (2000).

20. Ingwer Borg, P. J. F. G. *MDS Models and Measures of Fit*, 37–61 (Springer New York, New York, NY, 2005).

21. Pulido, A. *et al.* Functional materials discovery using energy-structure-function maps. *Nature* **543**, 657–664 (2017).

22. Case, D. H., Campbell, J. E., Bygrave, P. J. & Day, G. M. Convergence properties of crystal structure prediction by quasi-random sampling. *J. chemical theory computation* **12**, 910–924 (2016).

23. Price, S. L. *et al.* Modelling organic crystal structures using distributed multipole and polarizability-based model intermolecular potentials. *Phys. Chem. Chem. Phys.* **12**, 8478–8490 (2010).

24. Widdowson, D., Mosca, M., Pulido, A., Cooper, A. & Kurlin, V. Average minimum distances of periodic point sets - fundamental invariants for mapping all periodic crystals. *MATCH Commun. Math. Comput. Chem.* **87**, 529–559 (2022).

25. Ropers, J., Mosca, M. M., Anosova, O., Kurlin, V. & Cooper, A. I. Fast predictions of lattice energies by continuous isometry invariants of crystal structures. In Pozanenko, A., Stupnikov, S., Thalheim, B., Mendez, E. & Kiselyova, N. (eds.) *Data Analytics and Management in Data Intensive Domains*, 178–192 (Springer International Publishing, Cham, 2022).

26. Liu, T.-Y. *et al.* Learning to rank for information retrieval. *Foundations Trends Inf. Retr.* **3**, 225–331 (2009).

27. Xie, T. & Grossman, J. C. Crystal graph convolutional neural networks for an accurate and interpretable prediction of material properties. *Phys. Rev. Lett.* **120**, 145301, DOI: 10.1103/PhysRevLett.120.145301 (2018).

28. Paszke, A. *et al.* Pytorch: An imperative style, high-performance deep learning library. *Adv. neural information processing systems* **32** (2019).

29. Abadi, M. *et al.* Tensorflow: a system for large-scale machine learning. In *Proceedings of the 12th USENIX Conference on Operating Systems Design and Implementation*, OSDI'16, 265–283 (USENIX Association, USA, 2016).
